# Factors associated with self-reported mental health difficulties in Somalia: a national health and demographic survey analysis of populations aged 10 years and older

**DOI:** 10.1080/16549716.2026.2727758

**Published:** 2026-09-02

**Authors:** Hibo Abdilahi Omer, Khadar Mowlid Abdi, Abdisalam Hassan Muse

**Affiliations:** a School of Dentistry, Amoud University, Borama, Somalia; b Faculty of Business and Economics, Amoud University, Borama, Somalia; c Research and Innovation Center, Amoud University, Borama, Somalia

**Keywords:** Mental health, disability, SHDS, multilevel analysis, chronic illness

## Abstract

**Background:**

Mental health is a neglected public health issue in post-conflict zones like Somalia.

**Objective(s):**

Given the absence of community-based data in Somalia, this study sought to determine the prevalence and associated factors with Self-Reported Mental Health Difficulties among individuals aged 10 years and older by utilizing a nationally representative sample.

**Methods:**

We analyzed secondary data from the 2020 Somali Health and Demographic Survey of 49,389 individuals aged 10 years and older. We used descriptive statistics and multilevel logistic regression models to estimate prevalence and pinpoint socioeconomic and clinical factors.

**Results:**

The overall prevalence of Self-Reported Mental Health Difficulties among Somali individuals aged 10 years and older was 0.83%. Multilevel analysis revealed that advanced age was a primary correlate, individuals aged 55 years and older had 14.30 times higher odds of reporting self-reported mental health difficulties compared with those aged 10–19 years. Significant clinical correlates included a history of stroke (AOR = 4.07), chronic back pain (AOR = 2.76), and high blood pressure (AOR = 2.57).

**Conclusions:**

While reported prevalence is relatively low, likely reflecting severe impairment and cultural under-reporting, the burden is disproportionately high among elderly individuals and those with physical comorbidities. Policy interventions should focus on secondary prevention by integrating mental health screening into primary care for chronic diseases and developing specialized geriatric support services to bridge the treatment gap in Somalia.

## Paper Context

- **Main findings:** The overall prevalence of self-reported mental health difficulties in Somalia is low (0.83%), but the burden is disproportionately concentrated among older adults and individuals with chronic physical comorbidities.
- **Added knowledge:** This study identifies formal secondary education and traditional nomadic living as being associated with lower odds of experiencing mental health difficulties in the Somali context.
- **Global health impact for policy and action:** Public health strategies should prioritize integrating mental health screening into primary care management of chronic physical conditions and establishing geriatric and urban mental health support structures.

## Background

Mental health is conceptualized as a state of well-being in which an individual realizes their own abilities, can cope with the normal stresses of life, and contributes productively to their community [1,2]. Within this spectrum, common mental disorders (CMD)—which include depression, anxiety, and somatoform disorders—represent a significant public health challenge affecting individuals at all stages of life [3,4]. Anxiety is generally defined by excessive worry or fear that interferes with daily functioning, while depressive disorders are characterized by a loss of interest, persistent sadness, and cognitive impairments [5].

To conceptually ground the understanding of mental health in low-resource and post-conflict settings, this study adopts the Biopsychosocial Model alongside the World Health Organization’s (WHO) International Classification of Functioning, Disability, and Health (ICF) framework. The Biopsychosocial Model posits that mental health difficulties do not arise in isolation; rather, they stem from a complex, dynamic interaction between biological vulnerabilities (e.g. chronic physical illnesses, neurological conditions, aging), psychological processes (e.g. stress perception, emotional regulation), and social environment factors (e.g. urban living conditions, educational attainment, displacement, and community support networks). Complementing this, the ICF framework emphasizes that disability and mental health difficulties are not merely static personal attributes but outcomes of interactions between health conditions and contextual environmental or personal factors. In post-conflict environments, such as Somalia, biological stressors—such as a high burden of unmanaged chronic physical diseases—frequently compound psychological distress. Simultaneously, social determinants—such as rapid urbanization, low literacy levels, and the disruption of traditional nomadic community structures—play a pivotal role in shaping an individual’s mental well-being and daily functioning. Utilizing this integrated theoretical approach allows for a comprehensive evaluation of how biological comorbidities and socio-environmental structures jointly associate with self-reported mental health difficulties.

According to the International Classification of Functioning, Disability, and Health (ICF), mental health is not merely the absence of illness but is influenced by a complex interplay of biological limitations and environmental factors, such as social support and community attitudes [6,7]. On a global scale, neuropsychiatric conditions account for approximately 9.8% of the total burden of disease [3,8]. It is estimated that at least one in four people will be affected by a mental health problem at some point in their lives, yet nearly 12% of the global burden of disease originates from low-income countries where resources are most scarce [3,9]. Recent reports indicate that over 300 million people suffer from depression worldwide, with anxiety disorders affecting an additional 264 million [10,11]. Despite these staggering statistics, mental health has historically been neglected in favor of infectious diseases, leading to a ‘treatment gap’ that results in reduced productivity and increased years lost to disability, particularly in developing nations [1,12].

The prevalence and determinants of these disorders are highly variable and influenced by localized socioeconomic and physical stressors. For example, research among adults with physical disabilities in Saudi Arabia identified that moderate-to-severe disability is a strong predictor of psychological distress, with anxiety levels significantly linked to the presence of chronic illnesses [5,13]. Similarly, community-based studies in Kenya have highlighted how external factors, such as sexual violation, HIV-positive status, and job-related stress contribute to a 3.84% diagnostic prevalence of depression and anxiety [10,14]. These findings suggest that individuals with limited autonomy or those facing significant health-related stressors are at an increased risk of developing abnormal levels of psychological distress [5,15].

In Ethiopia, with an estimated 12% of the general population suffering from various mental illnesses [3,16,17]. Furthermore, research in urban centers like Addis Ababa and Jimma has shown that factors such as low average family income and unique cultural habits—specifically the chewing of Khat—are independent predictors of psychiatric morbidity [18,19].

Khat (*Catha edulis*) is a flowering plant native to the Horn of Africa, whose fresh leaves contain cathinone, a natural amphetamine-like stimulant. In Somalia and broader East Africa, habitual Khat chewing is a deeply entrenched sociocultural practice that has been widely linked to elevated psychological distress, sleep disturbances, financial strain, and exacerbation of psychiatric symptoms.

While hospital-based studies across the Horn of Africa provide preliminary insights into clinical populations, they do not accurately reflect the psychological status of the general resident population. This lack of representative data hinders the development of effective health protection policies and localized intervention programs [20,21]. Therefore, this study was intended to determine the magnitude of self-reported mental health difficulties and evaluate the associated socioeconomic and behavioral factors among individuals aged 10 years and older in Somalia to bridge this existing gap in public health knowledge.

## Methods

### Study setting and design

This study employed a secondary data analysis approach using a cross-sectional design, utilizing data from the Somali Health and Demographic Survey (SHDS) conducted in 2020. The SHDS is a nationally representative survey providing comprehensive data on health and demographic indicators across all regions of Somalia.

### Study population and sampling procedure

The SHDS 2020 employed a multi-stage cluster sampling design to ensure national representativeness. Data were collected via standardized questionnaires administered by trained fieldworkers. Our analysis included a total of 49,389 participants aged 10 years and older, participants for whom complete data on mental health status and relevant sociodemographic and clinical covariates were available.

## Operational definition

### Self-reported mental health difficulties

The outcome variable was derived from the single proxy question embedded in the SHDS 2020 disability module: ‘Does [Name] have difficulty or trouble with mental health, remembering, or concentrating?’ Responses were coded as a binary outcome, where a response of ‘Yes’ was coded as 1 (indicating self-reported mental health difficulties) and ‘No’ was coded as 0.

### Study variables

The primary outcome was ‘Self-Reported Mental Health Difficulties among individuals aged 10 years and older,’ categorized as a binary variable (yes/no). The explanatory variables were selected based on existing literature and available SHDS data. Sociodemographic variables included: Sex (male, female), Age group (10–19, 20–34, 35–54, 55+), Residence (urban, rural, nomadic), Educational level (no formal, primary, secondary+), and Marital status (ever married, never married). Clinical and behavioral factors included self-reported binary (yes/no) indicators for chronic conditions (high blood pressure, diabetes, heart disease, stroke, and chronic back pain) ascertained directly from individual responses within the SHDS health module.

### Data analysis method

Data were extracted from the SHDS 2020, cleaned, and analyzed in Stata. DHS special numeric response codes (8 = ‘Don’t know’ and 9 = ‘Refused’) were explicitly recoded as system missing, prior to binary recoding. To account for the complex, two-stage cluster sampling design of the SHDS 2020, statistical analyses were performed using Stata’s complex survey suite (svy). Primary Sampling Units (clusters, HV001), regional stratification (HV024), and normalized household sampling weights (HV005/1,000,000) were explicitly specified (svyset HV001 [pweight = svy_wt], strata (HV024) singleunit (scaled)). Descriptive statistics, including frequencies and weighted percentages, were used to summarize population characteristics. The frequentist Pearson chi-square test evaluated bivariate associations. Multilevel survey logistic regression models were fitted to estimate Adjusted Odds Ratios (AOR) and 95% Confidence Intervals (CI) across three sequential specifications: Null Model (cluster variance), Model I (individual covariates), and Model III (full model including residence). Model fitness was evaluated using the Akaike Information Criterion (AIC), and cluster variation was assessed via the Intra-class Correlation (ICC). All statistical tests were two-sided (*p* < 0.05).

## Results

In Table 1, a total weighted sample of 49,389 participants aged 10 years and older was analyzed. More than half of the participants were female (55.18%). The largest age cohort was 10–19 years (36.67%), followed by those aged 20–34 years (30.01%). Regarding residence, 47.23% of the participants resided in urban areas, while rural and nomadic residents accounted for 26.38% and 26.39%, respectively. A significant majority (69.57%) had no formal education. In terms of self-reported physical health conditions, high blood pressure was reported by 3.55% of the participants, followed by diabetes (1.55%), heart disease (0.58%), and stroke (0.14%). The overall weighted prevalence of Self-Reported Mental Health Difficulties was 0.83% (*n* = 400).

**Table 1.** Sociodemographic and clinical characteristics of theparticipants (*N* = 49,389).

Variable	Category	Frequency (n)	Weighted %
Sex	Male	22,136	44.82
	Female	27,253	55.18
Age group	10–19	18,113	36.67
	20–34	14,824	30.01
	35–54	10,630	21.52
	55+	5822	11.79
Residence	Urban	23,326	47.23
	Rural	13,027	26.38
	Nomadic	13,036	26.39
Education	No formal	34,361	69.57
	Primary	8592	17.40
	Secondary+	6436	13.03
Chronic conditions	Blood pressure (yes)	1753	3.55
	Diabetes (yes)	766	1.55
	Heart disease (yes)	288	0.58
	Stroke (yes)	70	0.14
Prevalence	Mental health issue (yes)	400	0.83

As demonstrated in Table 2, several factors were significantly associated with Self-Reported Mental Health Difficulties. Participants who were never married reported a higher proportion of difficulties (1.06%) compared to those ever married (0.62%), *p* < .001. Urban residents (0.99%) had a significantly higher prevalence of self-reported difficulties than nomadic residents (0.35%), *p* < .001. Furthermore, self-reported chronic physical conditions were strongly associated with mental health status (*p* < .001), with the highest prevalence observed among participants with a history of stroke (7.14%) and high blood pressure (3.08%).

**Table 2.** Bivariate association between sociodemographic/clinical factors and mental health status.

Variable	Mental issue (yes) n (%)	Mental issue (no) n (%)	χ2\chi^2χ2	*p*-Value
Marital status			29.34	<0.001
Ever married	176 (0.62%)	28,144 (99.38%)		
Never married	224 (1.06%)	20,845 (98.94%)		
Residence			46.27	<0.001
Urban	231 (0.99%)	23,095 (99.01%)		
Nomadic	46 (0.35%)	12,990 (99.65%)		
Chronic illness				
Blood pressure	54 (3.08%)	1699 (96.92%)	116.63	<0.001
Diabetes	18 (2.35%)	748 (97.65%)	22.96	<0.001
Stroke	5 (7.14%)	65 (92.86%)	34.99	<0.001

Table 3 details the multilevel survey logistic regression analysis accounting for complex sampling design features. Sex was evaluated as a covariate, with females showing no statistically significant difference in odds compared to males (AOR = 1.05, 95% CI: 0.81–1.35). Age was strongly and progressively associated with Self-Reported Mental Health Difficulties. Compared to adolescents aged 10–19 years, the odds were higher among young adults aged 20–34 years (AOR = 2.92, 95% CI: 2.06–4.12), increasing further among those aged 35–54 years (AOR = 6.51, 95% CI: 4.28–9.89), and highest among adults aged 55 years and older (AOR = 14.30, 95% CI: 9.09–22.52). Attaining secondary education or higher was associated with significantly lower odds of reporting difficulties compared to having no formal education (AOR = 0.30, 95% CI: 0.19–0.46).

**Table 3.** Multilevel logistic regression of factors associated with mental issues.

Predictors	Null model	Model I (individual) AOR (95% CI)	Model III (full model) AOR (95% CI)
Sex *(Ref: male)*			
Female	–	1.08 (0.84–1.39)	1.05 (0.81–1.35)
Age Group *(Ref: 10–19)*			
20–34	–	2.98 (2.11–4.21)***	2.92 (2.06–4.12)***
35–54	–	6.84 (4.52–10.35)***	6.51 (4.28–9.89)***
55+	–	16.28 (10.38–25.52)***	14.30 (9.09–22.52)***
Education *(Ref: No formal)*			
Primary	–	0.82 (0.58–1.16)	0.85 (0.60–1.20)
Secondary and above	–	0.33 (0.21–0.50)***	0.30 (0.19–0.46)***
Clinical Factors			
High Blood Pressure *(yes)*	–	2.80 (1.99–3.94)***	2.57 (1.82–3.61)***
Stroke *(yes)*	–	4.35 (1.68–11.26)**	4.07 (1.54–10.77)**
Chronic vack pain *(yes)*	–	3.02 (1.50–6.04)**	2.76 (1.35–5.62)**
Residence *(Ref: urban)*			
Rural	–	(Not included)	0.78 (0.54–1.12)
Nomadic	–	(Not included)	0.21 (0.13–0.33)***
Random effects			
Cluster variance (sigma^2^)	0.382	0.231	0.135
ICC (%)	10.42%	6.58%	3.95%
AIC	4410.2	4292.6	4249.2

Note: *p < 0.05, **p < 0.01, ***p < 0.001. AOR = Adjusted Odds Ratio.

## Discussion

In this nationally representative analysis of the 2020 Somali Health and Demographic Survey (SHDS), the overall prevalence of self-reported mental health difficulties among adults was 0.83%. This figure is strikingly lower than estimates reported in neighboring East African settings, such as the 3.84% reported in the 2022 Kenya Demographic and Health Survey or the 10.04% reported in community-based studies in Ethiopia. However, rather than indicating a genuinely low clinical prevalence of mental illness in Somalia, this 0.83% figure must be interpreted as reflecting severe measurement limitations, deep-seated cultural stigma, and health system gaps. The use of a single-item, self-reported proxy question in a large demographic survey tends to capture only the most visible or severe functional impairments, missing milder or moderate psychological distress. Furthermore, pervasive social stigma surrounding mental health in post-conflict Somalia often leads to deliberate under-reporting by respondents. Therefore, this low prevalence represents the ‘tip of the iceberg,’ capturing individuals experiencing significant functional limitations rather than the true epidemiological burden of common mental disorders in the general population [16].

A key finding of this study was the profound association between age and Self-Reported Mental Health Difficulties. Individuals aged 55 and older were 14.30 times more likely to experience difficulties compared to the 10–19 age group (AOR = 14.30, 95% CI: 9.09–22.52).This finding is concordant with global trends suggesting that the elderly face a ‘double burden’ of biological cognitive decline and social isolation. In a post-conflict society like Somalia, the elderly may also carry the cumulative weight of decades of trauma, which manifests as chronic Self-Reported Mental Health Difficulties in later life. This is consistent with research in Saudi Arabia, which identified age-related chronic illness as a primary driver of psychological distress [5].

This study revealed a significant bidirectional link between physical and mental health. Participants with a history of stroke, chronic back pain, and high blood pressure had significantly higher odds of Self-Reported Mental Health Difficulties. This association is consistent with findings in Kenya, where chronic medical illness was a leading determinant of depression and anxiety [10]. The probable mechanism for this association involves the dysregulation of the hypothalamic–pituitary–adrenocortical axis and the sympathetic nervous system due to chronic physical stress [10,14]. Furthermore, the loss of autonomy and the financial strain associated with managing chronic conditions in a resource-limited setting likely exacerbate psychological morbidity [5].

Education emerged as a powerful associated with lower odds; those with secondary education or higher were 70% less likely to report Self-Reported Mental Health Difficulties. Education likely enhances health literacy and provides individuals with superior cognitive and economic resources to cope with life stressors. This aligns with Ethiopian studies where low income and lack of formal education were independent predictors of psychiatric morbidity [18].

Interestingly, nomadic residents showed significantly lower odds of Self-Reported Mental Health Difficulties compared to urban residents. This suggests that the traditional social fabric and communal support systems inherent in nomadic lifestyles may act as a buffer against mental health deterioration. Conversely, urban residents face stressors such as overcrowding, unemployment, and the erosion of traditional support networks, which are known to contribute to higher levels of psychological distress [1].

### Limitations

This study has several limitations. First, the cross-sectional nature of the SHDS data precludes the establishment of temporal or strict causal relationships between sociodemographic factors, chronic illness, and Self-Reported Mental Health Difficulties. Second, the measurement of Self-Reported Mental Health Difficulties relied on a single self-reported proxy question rather than validated clinical psychometric screening tools. Therefore, the data reflect perceived ‘Self-Reported Mental Health Difficulties’ rather than true clinical prevalence. Finally, deep-seated cultural stigma surrounding mental illness and a lack of diagnostic infrastructure likely lead to significant under-reporting.

## Conclusions

The prevalence of Self-Reported Mental Health Difficulties in Somalia is 0.83%, with the burden disproportionately affecting the elderly and those with chronic physical comorbidities. While the prevalence appears low, it likely represents the ‘tip of the iceberg’ regarding severe mental impairment. Factors, such as advanced age, stroke, hypertension, and urban residence, were significantly associated with higher odds, while higher education and nomadic living were associated with lower odds. These findings pose a significant challenge to the Somali healthcare system, which must now address the intersection of physical and mental health to improve the overall quality of life for its citizens.

## Data Availability

The datasets analyzed during the current study are available in the Somalia Microdata Repository and can be accessed via https://microdata.nbs.gov.so/index.php/catalog/50

## Preprints

This manuscript has not been published as a preprint in any repository.
